# How Interpersonal Justice Shapes Legitimacy Perceptions: The Role of Interpersonal Justice Trajectories and Current Experience

**DOI:** 10.3389/fpsyg.2020.582327

**Published:** 2020-10-23

**Authors:** Juan Liang, Bibo Xu

**Affiliations:** Department of Psychology, Hubei University, Wuhan, China

**Keywords:** perceived legitimacy, interpersonal justice/fairness, trajectory, current experience, dynamic

## Abstract

Despite a growing body of research on the relationship between justice and perceptions of an authority’s legitimacy, few studies have addressed the effects of changes in justice on perceived legitimacy. In the present study, we tested a dynamic model emphasizing the interactive influences of both interpersonal justice trajectories and current experience predicting perceived legitimacy. We tested the trajectory of interpersonal justice over time as a predictor of perceived legitimacy (Study 1) and the current experience of justice as a moderator of this link (Study 2). In Study 1 participants were randomly assigned to receive either improving or declining feedback from an anonymous tutor over the course of four days. Results showed that participants with an improving trajectory perceived the authority to have higher legitimacy. In Study 2 participants rated the tutor’s fairness on 3 consecutive weeks, which were used to identify naturally interpersonal trajectories; we then manipulated the current interpersonal justice experience in the fourth week. Results showed that the trajectory effect was significant when the current experience was just, but not when it was unjust.

## Introduction

Scholars have accumulated considerable evidence of links between justice and perceptions of an authority’s legitimacy (e.g., Tyler and Jackson, 2014; Wolfe et al., 2016). Perceived legitimacy—the belief that the actions of an authority are appropriate and proper (Suchman, 1995; Tyler, 2006)—is critical to cooperation with authorities and engagement within the groups (Tyler and Jackson, 2014). Despite the wealth of research focused on issues of justice and perceived legitimacy, little is known about the temporal dynamics of justice—that is, the relation between justice experienced over time and later perceived legitimacy.

Time plays an important role in the link between the experience of justice and the perception of the authority’s legitimacy. Individuals construct their legitimacy perceptions during their daily interactions with the authority over time (Tost, 2011). The process of forming legitimacy perceptions can be regarded as a form of social exchange (Blau, 1964) between the individual and the authority. These exchanges are reciprocal (Liang and Li, 2019) and recur over time (Colquitt and Zipay, 2015). Moreover, the individual’s experience of justice can fluctuate during individual-authority interactions over time, and this change in information is meaningful to the individual (Hausknecht et al., 2011; Rubenstein et al., 2019). Recent advances suggest that justice trajectories (i.e., changes in the experience of fairness over time) exhibit a unique influence on individuals’ attitudes and behaviors, such as job satisfaction and organizational commitment (e.g., Hausknecht et al., 2011; Rubenstein et al., 2019). The current study aims to improve our understanding of how justice trajectories affect the individual’s perception of the authority as legitimate.

In this study, we focus on a specific form of justice, namely interpersonal justice. This form of fairness is characterized by the authority’s expressions of dignity, politeness, and respect (Bies and Moag, 1986). Compared to distributive justice (fairness with regard to an outcome) and procedural justice (fairness with regard to the process by which the outcome was determined), interpersonal justice may be more strongly related to attitudes toward the authority (Colquitt and Zipay, 2015). This may be especially true in eastern cultures, where people tend to be more sensitive to interpersonal relationships in interactions with the authority, compared to their counterparts in the west (Chen et al., 2014; Zhu and Akhtar, 2014).

### Effects of Interpersonal Justice Trajectories on Perceived Legitimacy

Why should interpersonal justice impact perceived legitimacy? Following the logic of social exchange theory, when an authority treats people with respect and dignity (i.e., interpersonal justice), people may feel that they have a high-quality relationship with the authority, which represents a kind of exchange resource (Basu and Green, 1997; Tyler, 1997; Colquitt et al., 2012; Liang and Li, 2019). In order to maintain and strengthen this high-quality relationship (Mitchell et al., 2012; Chen et al., 2014), individuals may reciprocate with compliance to the authority (Tyler, 1996; Colquitt et al., 2012; Liang and Li, 2019). With repeated exchanges, there is increasing investment (Cropanzano and Mitchell, 2005; Fortin et al., 2014; Rubenstein et al., 2019), and the relationship matures over time (Blau, 1964; Colquitt et al., 2013). Thus, there should be an interpersonal justice trajectory across exchange episodes (Hausknecht et al., 2011; Rubenstein et al., 2019), with corresponding changes in perceived legitimacy.

**FIGURE 1:**
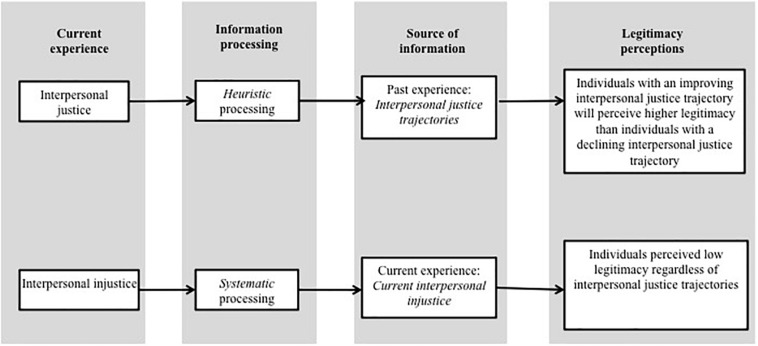
Proposed theoretical model of the interaction effect of justice trajectories and the current experience of interpersonal justice on perceptions of legitimacy.

We propose that interpersonal justice trajectories may provide independent information that is useful for predicting future perceived legitimacy. Specifically, we assume that individuals will evaluate an improving interpersonal justice trajectory as a signal of the authority’s increasing contributions to the exchange relationship, such as increased respect and care (Rubenstein et al., 2019). This evaluation may induce individuals’ feelings of gratitude and indebtedness, which strengthen the reciprocal interpersonal relations (Colquitt et al., 2007; Chen et al., 2014) and increase compliance to authority, thus strengthening the perception that the authority is legitimate (Tyler, 1996; Colquitt et al., 2012; Liang and Li, 2019). Conversely, a declining interpersonal justice trajectory could suggest that the individual’s situation is becoming progressively bleaker (Lindsley et al., 1995; Ariely and Carmon, 2000). As a result, the individual may become less invested in the social exchange relationship and withhold compliance to the authority (Blau, 1964; Colquitt et al., 2013; Rubenstein et al., 2019), whom they see as having low legitimacy (see Figure 1).

Hypothesis 1: Interpersonal justice trajectories explain variance in perceived legitimacy of the authority. Compared to a declining trajectory of interpersonal justice, an improving trajectory would predict higher perceived legitimacy.

### Interaction Between Interpersonal Justice Trajectory and Current Experience

Assuming our results show that perceptions of legitimacy are driven, to some extent, by interpersonal justice trajectories, a new question becomes critical. Would evaluations of perceived legitimacy at the end point of the trajectory be influenced by current fair or unfair treatment? An information processing perspective is a helpful way to conceptualize this question. The perspective addresses the question of how we process different types of information in different ways. One key distinction is between systematic and heuristic processing (Chen and Chaiken, 1999). Systematic processing is conceptualized as an analytic orientation to form attitudes; heuristic processing is a more limited mode of information processing in which people form attitudes by invoking heuristics (Maheswaran and Chaiken, 1991). Our assumption is that the type of processing used to make sense of justice information depends both on the trajectory (improving or declining) and current experience (just or unjust action by the authority).

We argue that it is easier to process an act of interpersonal justice than an act of interpersonal injustice because fair treatment by an authority is consistent with individuals’ general expectations (Folger and Cropanzano, 2001; Caleo, 2016; Zapata et al., 2016; Koopman L. et al., 2019). This allows the individual to direct their limited attentional resources to other information, such as information based on previous experience (Lind, 2001; Chaiken and Ledgerwood, 2012; Zapata et al., 2016). Given this, we propose that when experiencing interpersonal justice, individuals may rely on their interpersonal justice trajectories as a heuristic to form legitimacy perceptions (Lind, 2001; Hausknecht et al., 2011; Chaiken and Ledgerwood, 2012). Based on this heuristic, individuals with an improving interpersonal justice trajectory will perceive higher legitimacy than individuals with a declining interpersonal justice trajectory.

By contrast, a current experience of interpersonal injustice typically violates the assumption that an authority will be fair, requiring individuals to devote attention to and systematically process the authority’s actions (Lind, 2001; Zapata et al., 2016; Barclay et al., 2017; Koopman L. et al., 2019). Negative events trigger more effort at sense-making than positive events do (Baumeister et al., 2001), requiring systematic processing rather than automatic processing (Mayer and Gavin, 2005; Roberson, 2006; Posten and Mussweiler, 2013). In this context, the individual cannot use heuristics based on past experience to form legitimacy perceptions. Specifically, when experiencing interpersonal injustice, individuals with improving interpersonal justice trajectories would have the same legitimacy perceptions as those of individuals with declining interpersonal justice trajectories. That is, based on the authority’s current unjust behavior, there would be low perceived legitimacy regardless of trajectory.

Thus, based on an information processing perspective, we make two related predictions about the role of current experience in the process by which interpersonal justice trajectories are associated with perceived legitimacy. When the current experience is one of interpersonal justice, we posit that individuals may form legitimacy perceptions through heuristic-based processing, with little attention paid to the authority’s current just or unjust act. In contrast, when the current experience is one of interpersonal injustice, individuals may form legitimacy perceptions through systematic processing with greater attention to the authority’s current just or unjust act. In both cases, the interpersonal justice trajectory will interact with the current experience to predict perceived legitimacy.

Hypothesis 2: Interpersonal justice trajectories will interact with the current experience of interpersonal justice in predicting perceptions of legitimacy. Specifically, individuals with a declining interpersonal justice trajectory will perceive lower legitimacy than individuals with an improving interpersonal justice trajectory when the current experience is one of interpersonal justice, but not when the current experience is one of interpersonal injustice.

## Overview of Studies

We conducted two studies to test our hypotheses. Study 1 tested Hypothesis 1, and Study 2 tested Hypothesis 2. In Study 1 we manipulated the interpersonal justice trajectory over the course of four days (improving or declining) and then assessed participants’ perceptions of the authority’s legitimacy. In Study 2, we first identified naturally occurring groups (participants who reported experiencing increasing vs. decreasing justice over the course of 3 weeks); we then manipulated the current interpersonal justice experience, and assessed perceived legitimacy in the fourth week. We conceptualized legitimacy as “voluntarily deference to the authority,” which is one of the most common findings in the literature (Treviño et al., 2014; Tyler and Jackson, 2014). People complying with authorities voluntarily show that they accept and legitimate the authorities (Ponsaers, 2015).

### Study 1

Study 1 was designed to test whether interpersonal justice trajectories affect perceived legitimacy of the authority. This was a four-phase study over four consecutive days. The participants took part in the experiment at the same time every day. To manipulate the interpersonal justice trajectory, participants were randomly assigned to one of two conditions. In the “improving” trajectory group, participants were given feedback that was initially unjust but became increasingly just over the course of the four days. In the “declining” trajectory group, the feedback transitioned from being just to being unjust. The dependent variable was perceived legitimacy of authority.

#### Method

##### Participants

We recruited 74 college students (48 females; M *age* = 19.43, *SD* = 0.81) from undergraduate public courses and psychology courses at a large university in central China. We sought to make the sample size equivalent to that in previous research (e.g., Treviño et al., 2014, about 20–22 per cell; Jones and Skarlicki, 2005, about 17–20 per cell; Okimoto and Wenzel, 2009, about 25 per cell). We informed the college students during recruitment that the experiment would last for four days, so they should consider their schedules when determining whether to participant in this experiment. All participants provided informed written consent. Participants were informed during recruitment that the purpose of the study was to examine interpersonal relationships. Study 1 used a between-groups design (interpersonal justice trajectory: improving, declining). Participants were randomly assigned either to the improving interpersonal justice trajectory condition (*n* = 37) or the declining interpersonal justice trajectory condition (*n* = 37). The study was approved by the institutional review board (ethics committee) of the Faculty of Education at Hubei University. Participants were given a small present for their participation.

##### Procedure and materials

**FIGURE 2:**
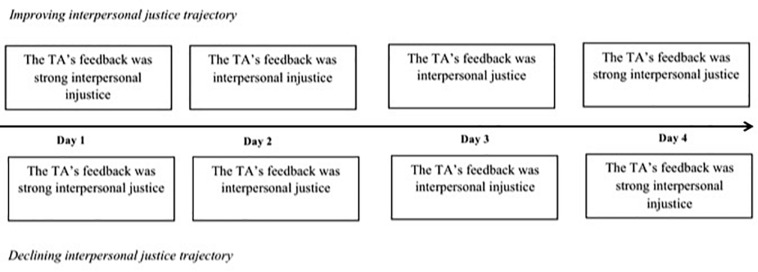
Summary of Study 1 Design.

Ten research assistants (RA) approached participants to explain the details of the experiment. The RAs contacted participants to remind them to participate in the experiment in a quiet room. Participants were asked to write a propositional essay for up to 45 min once a day for 4 days, and to email their propositional essay to an anonymous teaching assistant (TA) who would give feedback on the writing via email. The manipulation of the level of interpersonal justice was included in the TA’s scripted feedback at every time point (Okimoto, 2009; van der Toorn et al., 2011). Participants assigned to the improving interpersonal justice trajectory condition received feedback that progressed from *interpersonal injustice* to *interpersonal justice* over the course of the four days. Participants in the declining interpersonal justice trajectory condition received feedback of similar length and content, but it progressed from *interpersonal justice* to *interpersonal injustice* over the course of the four days (see Appendix A). After the manipulation, participants completed an online questionnaires assessing two types of manipulation check questions, one on each of the four days and one on the fourth day. Participants completed an online questionnaire to assess perceived legitimacy of the authority at Day 4. Then participants were debriefed and queried about the deception (see Figure 2).

We checked the effectiveness of the trajectory manipulation by carrying out two one-way ANOVAs, one for each item used to check the interpersonal justice trajectory manipulation (Manipulation check 1). Then, to checked the effectiveness of the trajectory manipulation repeatedly, we conducted A 2 (interpersonal justice trajectory: improving, declining) × 4 (Day: Day 1, Day 2, Day 3, and Day 4) repeated-measures ANOVA (RM-ANOVA) with the measure of interpersonal justice as the dependent variable (Manipulation check 2).

To determine whether the interpersonal justice trajectory manipulation influenced the scores for perceived legitimacy (Hypothesis 1), we used a one-way analysis of covariance (ANCOVA) with the assigned interpersonal justice trajectory as the between-subjects factor, the perceived legitimacy score as the dependent measure, and the perceived interpersonal justice score (measured at Day 4) as the control variable.

##### Measures

All survey items were assessed on a 5-point Likert scale (1 = *strongly disagree* to 5 = *strongly agree*).

##### Manipulation check: interpersonal justice

We checked the effectiveness of the interpersonal justice manipulation by asking participants to what extent they agreed with the statements developed by Colquitt (2001), at four time points: “*The teaching assistant treated me with patience*,” “*The teaching assistant treated me with dignity*,” and “*The teaching assistant treated me with respect.*” Cronbach alpha coefficients were 0.88, 0.87, 0.87, and 0.91 for Day 1, Day 2, Day 3, and Day 4, respectively.

##### Manipulation check: interpersonal justice trajectory

We checked the effectiveness of the trajectory manipulation by asking participants to what extent they agreed with the statements “*The TA’s attitude has gradually changed from very unfair to very fair*,” and “*The TA’s attitude has gradually changed from very fair to very unfair.*” This manipulation check question was given at Day 4.

##### Perceived legitimacy of the authority’

The item was: “*I should voluntarily comply with the TA’s decisions*” (van der Toorn et al., 2011). Perceived legitimacy of authority measured at Day 4 was the dependent variable in all analyses.

### Results

#### Manipulation Check 1: Perceptions of the TA’s Fairness

The first one-way ANOVA showed that participants in the improving interpersonal justice trajectory condition agreed more with the statement that the TA’s attitude had gradually changed from very unfair to very fair (*M* = 3.84, *SD* = 0.96) than those in the declining interpersonal justice condition (*M* = 1.68, *SD* = 0.78), *F*(1, 72) = 112.94, *p* < 0.001, η_*p*_^2^ = 0.61. The other one-way ANOVA showed that participants in the declining interpersonal justice trajectory condition agreed more with the statement that TA’s attitude had gradually changed from very fair to very unfair (*M* = 3.14, *SD* = 1.44) than those in the improving interpersonal justice condition (*M* = 2.03, *SD* = 1.12), *F*(1, 72) = 13.71, *p* < 0.001, η_*p*_^2^ = 0.16. These results indicated that the interpersonal justice trajectory manipulation was effective.

### Manipulation Check 2: Assigned Trajectories and Participants’ Perceptions of Interpersonal Justice

The results of a RM-ANOVA yielded a significant interpersonal justice trajectory × day interaction, *F*(3, 216) = 46.32, *p* < 0.001, η_*p*_^2^ = 0.39. Under the improving interpersonal justice trajectory condition, participants’ interpersonal justice perceptions increased over time, *F*(3, 108) = 33.71, *p* < 0.001, η_*p*_^2^ = 0.48, whereas under the declining interpersonal justice trajectory condition, participants’ interpersonal justice perceptions decreased over time, *F*(3, 108) = 14.62, *p* < 0.001, η_*p*_^2^ = 0.29 (see Table 1). These results support the evidence from the first manipulation check by showing that the interpersonal justice trajectory manipulation was effective.

**TABLE 1:** Perceived interpersonal justice as a function of interpersonal justice trajectory and time (Study 1).

Time								
	Day 1		Day 2		Day 3		Day 4	
Interpersonal justice trajectory	*M*	*SD*	*M*	*SD*	*M*	*SD*	*M*	*SD*
Improving interpersonal justice trajectory	2.50	0.88	2.95	0.84	3.55	0.82	4.12	0.65
Declining interpersonal justice trajectory	3.85	1.20	3.50	1.20	3.16	1.03	2.76	0.98

*N = 74. Ratings of perceived interpersonal justice were made on a 5-point scale, with higher numbers indicating higher perceived interpersonal justice.*

#### Perceived Legitimacy of the Authority

To determine whether the interpersonal justice trajectory manipulation influenced the scores for perceived legitimacy (Hypothesis 1), we used a one-way analysis of covariance (ANCOVA) with the assigned interpersonal justice trajectory as the between-subjects factor, the perceived legitimacy score as the dependent measure, and the perceived interpersonal justice score (measured at Day 4) as the control variable. The result yielded a significant effect of the interpersonal justice trajectory manipulation on the scores for perceived legitimacy, *F*(1, 72) = 6.53, *p* < 0.05, η_*p*_^2^ = 0.08. Participants in the improving interpersonal justice trajectory condition perceived higher legitimacy of authority (*M* = 2.89, *SD* = 0.97) than those in the declining interpersonal justice condition (*M* = 2.57, *SD* = 0.87). The findings showed that an improving trajectory perceived the authority to have higher legitimacy, which supported Hypothesis 1. These findings supported the notion that interpersonal justice trajectories predict legitimacy perceived after controlling for end-state levels of interpersonal justice.

### Study 2

Study 2 was designed to test the interaction between the interpersonal justice trajectory and the current experience of justice, with the perceived legitimacy score as the dependent variable. This was a four-phase study over four consecutive weeks. Based on the first 3 weeks of fairness ratings, two naturally-occurring trajectory groups were identified, corresponding to perceptions of improving or declining fairness. At the fourth week, we used a vignette to manipulate the current experience of interpersonal justice, and then assessed the perceived legitimacy of the authority.

#### Method

##### Participants and procedure

A total of 117 undergraduate psychology majors (87 females; M *age* = 20.62, *SD* = 1.16) at a large university in central China were recruited to participate in this study. We advertised this study as an investigation of teacher-student relationships. Study 2 aimed to trace participants’ interpersonal justice trajectories. It was impossible for us to determine the sample size in advance, so we simply included everyone who responded to our recruitment advertisement. All participants provided informed written consent. The study was approved by the institutional review board (ethics committee) of the Faculty of Education at Hubei University. The procedure consisted of three phases. In the *first* phase, the participants were asked to complete an online questionnaire assessing perceptions of interpersonal justice once a week for 3 weeks.

In the *second* phase, we employed latent growth mixture modeling (LGMM) to identify naturally interpersonal justice trajectories based on the perceptions of interpersonal justice, which were collected at the first 3 weeks (Muthén and Muthén, 2000; Muthén, 2004). Based on the results of the LGMM analyses, 49 participants perceived an improving interpersonal justice trajectory, and 68 participants perceived a declining interpersonal justice trajectory. After that, participants were invited to participate in the vignette experiment at Time 4 (conducted 1 week after Time 3).

**FIGURE 3:**
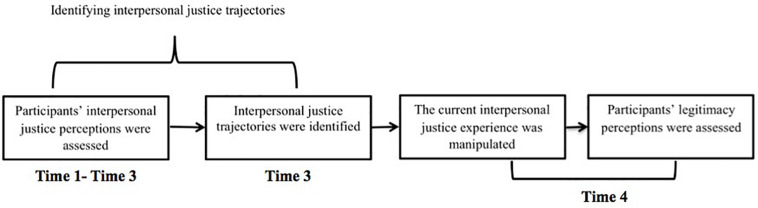
Summary of Study 2 Design.

In the *third* phase, we employed an online vignette design to manipulate the current interpersonal justice experience at the fourth week. All participants read a scenario and were asked to imagine that they had recently experienced the situation. Participants assigned to the current interpersonal justice condition (25 of whom perceived an improving interpersonal justice trajectory, and 31 of whom perceived a declining interpersonal justice trajectory, according to the results of LGMM in the second phase) read a scenario indicating that the tutor was currently behaving in a fair way interpersonally. Participants assigned to the current interpersonal injustice condition (24 of whom perceived an improving interpersonal justice trajectory, and 37 of whom perceived a declining interpersonal justice trajectory, based on LGMM) read a scenario of similar length and content but it indicated that the tutor was behaving in an unfair way interpersonally (see Appendix B) (see Figure 3).

##### Measures

All survey items were assessed on a 5-point Likert scale (1 = *strongly disagree* to 5 = *strongly agree*).

##### Perceived interpersonal justice

We assessed interpersonal justice with three items that were used in Study 1, at the first three time points: “*The tutor treated me patiently*,” “*The tutor treated me with dignity*” and “*The tutor treated me with respect.*” Cronbach alpha coefficient = 0.85, 0.92, and 0.89 for Time 1, Time 2, and Time 3, respectively. Scores on this measure at the three time points were used in the LGMM analysis to identify discrete growth class.

##### Manipulation check

We checked the effectiveness of the current interpersonal justice experience manipulation with three items by asking participants to what extent they agreed with the statements that “*The tutor treated me patiently in the scenario*,” “*The tutor treated me with dignity in the scenario*” and “*The tutor treated me with respect in the scenario*.” Cronbach alpha coefficient = 0.99. The manipulation check questions were asked at Time 4.

##### Perceived legitimacy of the authority

Perceived legitimacy of authority was measured at Time 4, using the same item used in Study 1: “*I should voluntarily comply with the tutor’s decisions*.” Perceived legitimacy of authority was the dependent variable in the analyses.

#### Statistical Analysis

We tested Hypothesis 2 in two steps. First, LGMM was used to identify latent classes of perceived interpersonal justice trajectories by using Mplus Version 7.2 (Muthén and Muthén, 2012). A critical component of LGMM is that it does not assume a single population and can test for the presence of multiple groups or classes of individuals that represent distinct multivariate normal distributions. These discrete populations are modeled using categorical latent variables (classes) in combination with continuous latent variables that define a particular growth trajectory within each class (for example, intercept and slope). To facilitate model specification, we compared one- to three-class unconditional LGMM models, using conventional indices to identify the model with superior fit.

Second, we used Mplus to save each participant’s trajectory factor scores (using the SAVE = CPROBABILITIES command). This command returns a value for each participant regarding his or her interpersonal justice trajectory class. We next used ANOVA to test the interaction between the LGMM trajectory group and the current experience of interpersonal justice in the prediction of perceived legitimacy (Hypothesis 2).

#### Results

##### Descriptive statistics

The means, standard deviations, reliabilities, together with the correlations among all variables, are shown in Table 2.

**TABLE 2:** Means, standard deviations, reliabilities, and correlation matrix of perceived interpersonal justice (Study 2).

	*M*	*SD*	1	2	3
(1) Time 1	4.33	0.52	(0.85)		
(2) Time 2	4.32	0.52	0.70**	(0.92)	
(3) Time 3	4.35	0.50	0.65**	0.85**	(0.89)

N = 117. Internal consistency reliabilities (Cronbach’s alphas) appear in parentheses along the diagonal. **p < 0.01, two-tailed.

##### LGMM to identify interpersonal justice trajectories

Table 3 presents the results of the LGMM by showing the fit indices for the solutions with different numbers of latent trajectory classes. To determine the appropriate class solution, we examined the Bayesian Information Criteria (BIC), Akaike Information Criteria (AIC), Adjusted BIC, and entropy values (Muthén, 2003; Nylund et al., 2007). We sought a model with lower values for the criterion indices and higher entropy values (Muthén, 2003; Nylund et al., 2007). The results showed that a two-class solution had the best fit to the data.

**TABLE 3:** Fit indices for growth mixture models of interpersonal justice with different number of latent classes (Study 2).

Number of classes	AIC	BIC	ABIC	Entropy
1	389.18	411.28	385.99	
2	306.32	336.70	301.93	0.99
3	353.99	392.66	348.40	0.85

Table 4 provides information about the two-class solution that was used in subsequent analyses. In this solution, the first latent trajectory class (*n* = 49) consisted of those participants with a significant pattern of growth in perceived fairness across time. This latent trajectory class was labeled “*improving interpersonal justice trajectory*.” The second latent trajectory class, “*declining interpersonal justice trajectory*” (*n* = 68), was characterized by a pattern of significant decreases in perceived fairness across time.

**TABLE 4:** Growth factor parameter estimates for 2-class model: Perceived interpersonal justice (Study 2).

Class	*n*	Intercept		Slope		*Post hoc* analyses
		Est.	SE	Est.	SE	
1	49	4.76	0.09	0.11	0.04	1.00
2	68	4.10	0.06	–0.07	0.03	1.00

*Est., Estimate; SE, standard error.*

##### Manipulation check

A 2 (interpersonal justice trajectory: improving, declining) × 2 (current interpersonal justice experience: justice, injustice). ANOVA showed a significant main effect of current interpersonal justice experience on perceived interpersonal justice, *F*(1, 113) = 403.01, *p* < 0.001, η_*p*_^2^ = 0.78. Participants in the current interpersonal justice condition perceived higher interpersonal justice (*M* = 4.60, *SD* = 0.81) than those in the current interpersonal injustice condition (*M* = 1.75, *SD* = 0.71). These results indicated that the current interpersonal justice experience manipulation was successful.

##### Perceived legitimacy of the authority

**FIGURE 4:**
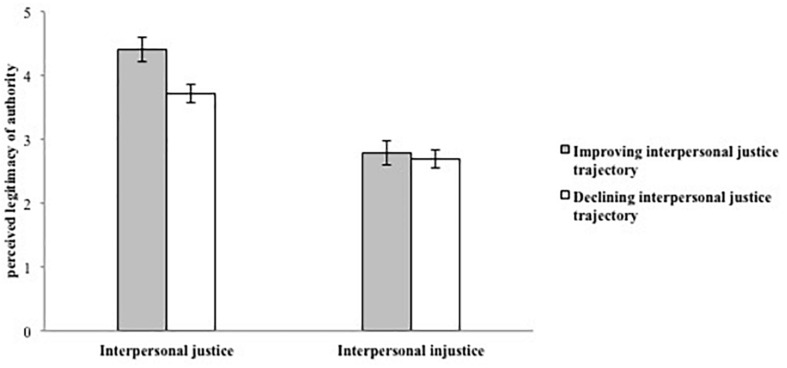
Perceived legitimacy of authority as a function of interpersonal justice trajectory and current experience (Study 2). Ratings of perceived legitimacy of authority were made on a 5-point scale, with higher numbers indicating higher perceived legitimacy.

Perceived legitimacy was used as the dependent measure in a 2 (interpersonal justice trajectory: improving, declining) × 2 (current interpersonal justice experience: justice, injustice) ANOVA. The main effect of interpersonal justice trajectory was significant, *F*(1, 113) = 7.37, *p* < 0.01, η_*p*_^2^ = 0.06. Participants in the declining interpersonal justice trajectory group reported lower perceived legitimacy (*M* = 3.16, *SD* = 0.92) than those in the improving interpersonal justice trajectory group (*M* = 3.61, *SD* = 1.10). The main effect of current interpersonal justice experience was also significant, *F*(1, 113) = 82.99, *p* < 0.001, η_*p*_^2^ = 0.42. Participants in the current interpersonal injustice experience condition reported lower perceived legitimacy (*M* = 2.74, *SD* = 0.89) than those in the current interpersonal justice experience (*M* = 4.02, *SD* = 0.67). Importantly, these effects were qualified by the hypothesized two-way interaction between interpersonal justice trajectory and current experience, *F*(1, 113) = 4.39, *p* < 0.05, η_*p*_^2^ = 0.04 (see Figure 4). As predicted, simple main effects indicated that among participants in the current interpersonal justice experience condition, those in the declining interpersonal justice trajectory group reported lower perceived legitimacy (*M* = 3.71, *SD* = 0.64) than those in the improving interpersonal justice trajectory group (*M* = 4.40, *SD* = 0.50), *F*(1, 113) = 7.11, *p* < 0.01, η_*p*_^2^ = 0.06. Among participants in the current interpersonal injustice experience condition, there was not a difference between the declining and improving trajectory groups in ratings of perceived legitimacy, *F*(1, 113) = 3.31, *p* > 0.05, η_*p*_^2^ = 0.03.

### General Discussion

In this study we explored how individuals use interpersonal justice trajectories to construct their perceptions of an authority’s legitimacy; we also investigated the interactive effect of interpersonal justice trajectories and the current experience of justice on these perceptions. Study 1 showed that improving (declining) interpersonal justice trajectory motivated higher (lower) legitimacy perceptions. The results of Study 2 showed that interpersonal justice trajectories and the current experience also interacted to predict perceived legitimacy. Among participants who were currently experiencing interpersonal justice, those who were in a declining interpersonal justice trajectory reported lower perceived legitimacy than those who were in an improving interpersonal justice trajectory. This effect was not obtained in the current interpersonal injustice condition. This study is important because it is the first to show that individuals draw upon both static (i.e., end-state justice treatment) and dynamic (i.e., justice trajectories) characteristics when forming legitimacy perceptions.

### Theoretical Implications

This research contributes to the legitimacy literature in several ways. First, whereas the relationship between justice and perceived legitimacy has been well documented (e.g., Tyler and Jackson, 2014; Wolfe et al., 2016), prior research focused on the link between the temporal events or entity reference justice perceptions and contemporaneous legitimacy perceptions (e.g., van der Toorn et al., 2011; Bradford, 2014; Tyler and Jackson, 2014; Tankebe et al., 2016). The present research extended these earlier investigations by first taking into account improvements or decrements (i.e., justice trends) over time in how individuals have been treated (i.e., current justice experience). This is an important consideration, as scholars have argued that people may develop their legitimacy perceptions of authority figures over the course of repeated interactions (Tost, 2011). In addition, legitimacy is a generalized perception on the authority (Sidani and Rowe, 2018). This generalized perception may be shaped by both static (e.g., end-state evaluations) and dynamic (e.g., trend over time) properties (Ariely and Carmon, 2003).

Second, we found that individuals’ negative interpersonal fairness trajectories may reduce legitimacy perceptions, even when the authority does enact justice in the end. When individuals experience interpersonal justice, they may rely on their interpersonal justice trajectories as a heuristic to form legitimacy perceptions. The negative trajectory implies that the authority will less invest into the social exchange relationship (Rubenstein et al., 2019). As a result, even though experiencing interpersonal justice, individuals with a negative interpersonal justice trajectory will hesitate to rate high legitimacy. This finding implies that the authority’s justice behavior might not always improve individuals’ perceptions of the authority’s legitimacy. This challenges the consensus that perceptions of legitimacy are improved by an authority’s justice enactments (e.g., Bradford, 2014; Tyler and Jackson, 2014; Tankebe et al., 2016). Moreover, the individual’s justice trajectory may provide a heuristic for evaluating legitimacy, one that disregards the current experience of justice.

The present results also contribute to theory and research concerning evaluations of fairness. Previous studies showed that individuals create heuristics based on social cues (Bianchi et al., 2015), based on one’s trust in the authority, one’s justice judgments and information from one’s peers (e.g., Lind, 2001; Jones and Skarlicki, 2005; De Cremer and Tyler, 2007; Bianchi et al., 2015). The results of the present study are consistent with the possibility that changes in the experience of justice over time created a heuristic that biased individuals’ perceived legitimacy.

Rubenstein et al. (2019) made a recent call to conduct research on the effect of justice on attitudes and behavior by modeling the present experience of justice in conjunction with past trajectories. Their own research met this goal with a focus distributive and procedural justice, and showed that the effect of present justice on employees’ helping behavior and voluntary turnover were shaped by justice trajectories. Specifically, the positive relationship between present justice level and helping behavior, and the negative relationship between present justice level and voluntary turnover, were stronger for employees with an improving justice trajectory than for those with a declining interpersonal justice trajectory.

Our own research also took into account both the present experience of justice and justice trajectories, but differed from Rubenstein et al.’s (2019) research in two respects. Specifically, we focused on interpersonal justice rather than distributive and procedural justice, and examined the justice effect on perceived legitimacy rather than behavior. We explicitly tested the interaction between current experience and interpersonal justice trajectory in experiments that manipulated both factors. In doing so, the present justice experience was independent of one’s past trajectories.

A key contribution of this study is that we use information processing perspective to conceptualize the interactive relationship between justice trajectories and later acts by the authority in predicting perceived legitimacy. Based on the information-processing perspective, we conclude that justice trajectories create a heuristic by which information is automatically processed without attention to current experience; the systematic processing of the current justice experience is less likely to occur.

Finally, two the methodological strengths deserve to mention. First, similar to recent research (Matta et al., 2017), we applied the within-individual approach to manipulate interpersonal justice in Study 1. This study design answers the calls for taking a within-individual perspective into justice phenomena (Koopman J. et al., 2019).

Second, when analyzing the interactive effect of justice trajectories and present justice perceptions, previous researchers assessed the justice perceptions at different time points, which were used to identify the justice trajectories; then they computed interaction terms for trajectories and last reported justice levels (Rubenstein et al., 2019). This design may raise questions about the impact of justice trajectories on the end-stated justice levels. To reduce this possibility, we manipulated the current interpersonal justice experience after identifying naturally occurring groups.

### Practical Implications

Given our findings that interpersonal justice trajectories influence individuals’ legitimacy perceptions, the authority may find it useful to consider individuals’ unique histories of perceived justice. Thus, to gain a higher legitimacy, the authority should be concerned about tracking individuals’ ongoing justice experiences over time. The ongoing assessment of attitudes toward the authority and toward justice, perhaps in the context of discussions during employee or student evaluations, may help to identify those individuals who have a downward spiral and who perceive low legitimacy. Second, this study elucidates an important dilemma for the authority: for individuals with a declining interpersonal justice trajectory, receiving fair treatment by an authority may not override the perception of low legitimacy. Thus, when interacting with those individuals, authorities need to consistently enact fair treatment to change the perception of a declining justice trajectory into the perception of an improving justice trajectory.

### Limitations and Future Directions

One limitation concerns the somewhat restricted variability in the justice trajectories based on participants’ ratings of justice over the course of 4 weeks in Study 2. That is, the justice ratings showed significant differences over time (making it possible to detect trajectories), but students made very similar changes from one time point to the next. Although it is unclear why this would happen, it could be related to the fact that the students were familiar with their tutors. Thus, future studies should consider other populations where justice levels are liable to show greater fluctuations, to bolster the generalizability of our findings. For instance, new members of a community or organization, who are uncertain about their status and belongingness, may be more sensitive to fluctuation in how fairly they are treated (e.g., van den Bos and Lind, 2002) and thus may show greater variability in their justice evaluations from one time point to another (De Cremer and Sedekides, 2005; Bradford, 2014).

Second, our study collected data about student-tutor relationships, raising a potential concern about generalization to other contexts. In the views of Chinese students, their tutors possess power to some extent. Hence, the tutors are viewed as influential authorities in the eyes of students. However, the power and influence of the authorities may vary widely across individual-authority relationships in differing social contexts. To increase generalizability, future research should examine whether the present findings generalize to social and legal contexts. In addition, this is the first study to link changes in interpersonal justice to perceived legitimacy. However, distributive and procedural justice are also key influences on perceptions and behavior (van der Toorn et al., 2011; Tyler and Jackson, 2014). Future research could build on our findings by linking these other justice dimensions, and their variation over time, to perceived legitimacy. For example, compared to procedural justice, the effects of distributive justice on perceived legitimacy may be more similar to those of interpersonal justice, as the procedures in the organization are stable.

Third, although we found that justice trajectories interact with current justice experience to influence perceived legitimacy, we were unable to directly test the reasons for this effect. For example, we regarded the process of developing legitimacy perceptions as due to a series of social exchanges over time, with perceived legitimacy as a resource in these exchanges; however, we did not directly measure perceptions of social exchange quality (Colquitt et al., 2013; Rubenstein et al., 2019). It will be useful in future research to test this and other possible moderators of the effect of interpersonal justice on perceived legitimacy. Relatedly, the potential impact of self-efficacy might be relevant to the findings of Study 1. Based on the relational models of justice (Tyler and Lind, 1992) and social exchange theory (Blau, 1964), the changes of participants’ self-efficacy, which might be induced by the TA’s feedback, may affect the participants’ sense of self. The change of sense of self may in turn affect participants’ attitudes and behaviors to the authorities (Tyler and Lind, 1992; De Cremer and Sedekides, 2005). For instance, when participants received feedback that progressed from positive feedback to negative feedback, their self-efficacy may decline. In order to protect from the negative self, the individual may become less invested in the social exchange relationship (Blau, 1964; Colquitt et al., 2013; Rubenstein et al., 2019). It is worth considering the role of self-efficacy when explaining the relationship between justice trajectory and legitimacy.

Fourthly, because participants in the injustice experience group in Study 2 showed low ratings on perceived legitimacy, we are unable to rule out alternative explanations such as people with current experience of injustice more biased than those with current experience of justice. That is, the current experience of injustice might have put people in a negative affective and cognitive state, which might lead people to make judgments in a biased way. Future research may focus on this alternative mechanism. Moreover, it may be advisable for researchers to control for negative state to provide a better test of the validity of our findings.

Finally, it should be noted that, our research was conducted in a single cultural context, which does not allow any test of the universality of our findings. Confucian values are deep-rooted in Chinese culture (Hwang, 2000). *Relationalism*, which refers to the principle of favoring intimates and close relationships, is one of the pillars of Confucianism (Hwang, 2000). The value of relationalism leads the Chinese to be more reliant on relationships (Chen et al., 2014). This suggests that the Chinese may be more concerned than westerners about the dignity and respect by the authority (i.e., interpersonal justice). Future research should therefore include samples from multiple nationals that differ on relationalism to test whether the influence of interpersonal justice trajectories on perceived legitimacy is specific to Chinese culture or not. In addition, research is needed in other nations, which share similar culture values with China, in order to examine our findings’ generalizability.

## Conclusion

In this research we add a dynamic perspective on perceptions of an authority’s legitimacy by examining how both interpersonal justice trajectories and the current experience of justice independently and interactively predict individuals’ legitimacy perceptions. Our findings indicate that interpersonal justice trajectories affect perceived legitimacy: individuals with an improving interpersonal justice trajectory over time perceive higher legitimacy than those with a declining interpersonal justice trajectory. Moreover, this effect is moderated by the current experience of just or unjust behavior. A declining interpersonal justice trajectory reduces individuals’ legitimacy perceptions, even when the authority does enact justice in the end.

## Data Availability Statement

The raw data supporting the conclusions of this article will be made available by the authors, without undue reservation.

## Ethics Statement

The study was approved by the Institutional Review Board (Ethics Committee) of the Faculty of Education at Hubei University. The patients/participants provided their written informed consent to participate in this study.

## Author Contributions

JL conceived and designed the experiments and analyzed the data. Both authors performed the experiments and wrote the manuscript.

## Conflict of Interest

The authors declare that the research was conducted in the absence of any commercial or financial relationships that could be construed as a potential conflict of interest.

## Appendix A: Manipulation in Study 1

Strong interpersonal injustice: Your article is not well written. Did you spend enough effort on it? With regard to the viewpoint, you presented a new perspective, but you only expressed the main idea instead of fully presenting your opinion. Your essay doesn’t reach the standard of an undergraduate! Interpersonal injustice: Your essay is not well written. With regard to the argument, your essay could reflect your thoughts, but you didn’t thoroughly expound your argument in the essay and only explained it simply. It’s obvious that you didn’t finish your task earnestly! Interpersonal justice: Hello. With regard to the logic, your article could reflect your ideas, but it didn’t present your understanding of the practice itself. It seems that you just simply listed your arguments. Please make some changes, it will improve the quality of your article. Strong interpersonal justice: Hello. After reading your article, I can tell that you spared no efforts to finish the essay. From a structural point of view, the framework was presented. But some ideas were not expressed, as they were limited by the former structure. Please revise your article to make it better. The operationalization of *improving interpersonal justice trajectory* was feedback in the following order: strong interpersonal injustice level→ interpersonal injustice level→ interpersonal justice level→ strong interpersonal justice level. The operationalization of *declining interpersonal justice trajectory* was feedback in the following order: strong interpersonal justice level→ interpersonal justice level→ interpersonal injustice level→ strong interpersonal injustice level.

## Appendix B: Manipulation in Study 2

Please imagine you are the target person in the scenario: You met some difficulties while writing your graduation thesis, you had to discuss it with your tutor face to face urgently. Current interpersonal justice: Your tutor stopped his/her work when you met with him/her. During the discussion, he/she was KIND HEARTED and PATIENT. Current interpersonal injustice: Your tutor kept doing his/her work when you met with him/her. During the discussion, he/she BEHAVED COOLLY and IMPATIENTLY.
